# The Acute Relationship between Affective States and Stress Biomarkers in Ethnic Minority Youths

**DOI:** 10.3390/ijerph182312670

**Published:** 2021-12-01

**Authors:** Cheng K. Fred Wen, Chih-Ping Chou, Britni R. Belcher, Marc J. Weigensberg, David S. Black, Donna Spruijt-Metz

**Affiliations:** 1 Center for Self-Report Science, University of Southern California, Los Angeles, CA 90089, USA; 2 Department of Population and Public Health Sciences, University of Southern California, Los Angeles, CA 90032, USA; cchou@usc.edu (C.-P.C.); bbelcher@usc.edu (B.R.B.); davidbla@usc.edu (D.S.B.); 3 Department of Pediatrics, University of Southern California, Los Angeles, CA 90033, USA; weigensb@usc.edu; 4 Center for Economic and Social Research and Department of Psychology, University of Southern California, Los Angeles, CA 90089, USA; dmetz@usc.edu

**Keywords:** youth, emotion, cortisol, dietary behavior, physical activity, hypothalamic–pituitary–adrenal axis, obesity, ethnic minority

## Abstract

*Background*: Whether affective states acutely predict the hypothalamic–pituitary–adrenal (HPA) axis activities and whether energy balance-related behaviors moderate the affect–HPA axis relationship in obese youths are not well-understood. *Methods*: 87 mostly obese (94.3% obese) minority adolescents (mean: 16.3 ± 1.2 years old; 56.8% Latino and 43.2% African American) participated in a randomized crossover trial in an observation laboratory, where they received either high-sugar/low-fiber (HSLF) or low-sugar/high-fiber (LSHF) meals first and then crossed over in the next visit 2–4 weeks later. During each visit, they rated five affective states and provided a saliva sample every 30 min for the first 5 h and wore a waist-worn accelerometer. The association between the affect ratings and cortisol levels in the subsequent 30 min and the moderation effect of energy balance-related behavior were examined using multilevel models. *Results*: Within-person negative affect (β = 0.02, *p* = 0.0343) and feeling of panic (β = 0.007, *p* = 0.004) were acutely related to the subsequent cortisol level only during the HSLF condition. The time spent in moderate-to-vigorous physical activity did not moderate the acute relationship between affect and the subsequent cortisol level. *Conclusions*: Negative affect could be acutely related to heightened HPA axis activities in youths, but only when they were exposed to meals with high sugar and low fiber content. These results suggest that the meals’ sugar and fiber content may modulate HPA axis reactivity to negative affect in youths.

## 1. Introduction

The hypothalamic–pituitary–adrenal (HPA) axis is an important neuroendocrinological system that provides physiological resources for the human body when faced with psychological and physiological demands that could challenge homeostasis [1]. Repeated activation of the HPA axis due to prolonged exposure to psychological or physiological demands can lead to the development of a dysregulated and maladaptive HPA axis [2,3]. The increase in exposure to cortisol, an indicator of HPA axis activation, has been linked to decreased insulin sensitivity [4], a major risk factor for developing type II diabetes [5], as well as increased risks of various adverse mental and physical health conditions [6,7]. Identifying predictors of and mechanisms that could acutely lead to increased cortisol exposure could provide future research with valuable insights on viable intervention targets for preventing individuals from exposure to a heightened level of cortisol and further developing other related adverse health outcomes.

Affective states are possible acute predictors of increased cortisol levels. Affective states are momentary experiences or feelings that an individual has about an actual or perceived internal or external event [8,9]. These feelings could present psychological demands and thereby activate the HPA axis, as indicated by an increased level of cortisol. An early meta-analysis showed that compared to the control groups, adults exposed to experimental conditions designed to elicit various feelings (e.g., fear of losing social approval, rumination, threats, etc.) had increased cortisol levels as early as 30 min after exposure to the experimental condition [10]. Evidence of acute HPA axis reactions to affective states in adolescents, however, is limited. During childhood and adolescence, many psychological and physiological functions develop and change [11,12], including HPA axis reactivity to stressors [12]. During adolescence, youths encounter various new experiences and feelings. Several studies showed that on days when youths reported experiencing higher negative affect, their cortisol levels also declined at a slower rate throughout the day [13,14,15,16], indicating elevated HPA axis activities (for the review, see [7]). Laboratory-based studies have shown that emotion induction and laboratory-induced emotional stressors lead to noticeable changes in cortisol levels as early as 30 min after the exposure [10]. However, whether affective states that occur naturally (i.e., unstimulated affective states) are related to cortisol levels on a momentary basis remains unresolved. Investigating the acute HPA axis reactivity to unstimulated affective states in adolescence may provide valuable targets for intervention that focus on reducing exposure to cortisol. Such investigation could be especially important for overweight and obese adolescents who are at an elevated risk for experiencing weight-related victimization [17] that could translate into an elevated level of negative affect, as well as the development of related adverse health outcomes [6,7]. Therefore, the first aim of this study was to examine whether affective states are acutely related to salivary cortisol levels in obese ethnic minority youths.

The substantial between- and within-person variations in the effects of affective states on HPA axis activities [10,18] suggest that there may be unexplored moderators in the affect–HPA axis relationship. Energy balance-related behaviors, including moderate-to-vigorous physical activity (MVPA) and dietary behavior, could be possible behavioral moderators in this relationship. Both MVPA and dietary behavior have been shown to change affective states acutely. Recent evidence has suggested that MVPA could acutely improve youths’ affective states [19,20]. Furthermore, recent evidence has shown that participation in physical activity of moderate intensity reduced adults’ HPA reactivity to stressors [21,22]. On the other hand, dietary behavior, such as breakfast consumption [23] and sugar consumption [24,25], has been shown to affect individuals’ affective states. While acute changes in eating behavior after being exposed to stressors were reported by various prior studies [26,27], whether dietary behavior could modify how affective states relate to cortisol levels has rarely been discussed. Understanding the role of these energy balance-related behaviors in modifying the affect–HPA axis relationship could provide valuable targets for prevention and intervention efforts that aim to reduce the risk of developing adverse health outcomes related to a maladaptive HPA axis. However, whether these behaviors could acutely change how affective states relate to HPA axis activities remains unexplored. The second aim of this study was to examine the potential moderating role of MVPA and sugar consumption in the acute predictive relationship between affective states and HPA axis activities.

## 2. Materials and Methods

### 2.1. Study Sample

The data for this study came from a laboratory-based randomized crossover trial that aimed to examine the effect of meal contents on ethnic minority adolescents’ mood states, physical activity, and metabolic indices. The full study procedure that includes details that pertain to the information aligned with the CONSORT guidelines for randomized crossover trials [28], including condition randomization, is reported in detail elsewhere [29]. Briefly, this study is a secondary data analysis from a randomized crossover trial where the participants visited the study observation laboratory on two separate days that were 2–4 weeks apart. For the first visit, each participant was randomized into one of the two meal conditions, high sugar and low fiber (HS) or low sugar and high fiber (LS), followed by an 8 h in-laboratory observation period. Each participant returned to the laboratory for the second time and received the other meal condition after at least two weeks of the washout period, followed by another 8 h in-laboratory observation period. The HS breakfast contained 41.0 g of sugar and 1.0 g of fiber, whereas the LS breakfast contained 7.0 g of sugar and 16.0 g of fiber [29]. The study participants were recruited from the Los Angeles area (born in 2007–2010). The inclusion criteria were as follows: adolescents aged between 14 and 18 years old, of African American or Latino ethnicity, and with a body mass index ≥85th percentile for age and sex. The exclusion criteria included diagnosis of diabetes, participation in a weight loss or exercise program, use of medications that influenced body weight or insulin sensitivity, or diagnosis of a syndrome that influences body composition.

### 2.2. Study Procedure

After signed parental consent and participant assent were provided, the study participants were scheduled for two visits at the university observational laboratory, each after a 10 h overnight fast. Each participant received experimental breakfast and lunch meals (either HS or LS condition for the first visit and the other condition at the second visit) and was otherwise instructed to engage freely in activities available in the observation laboratory throughout their 8 h stay. The naturalistic observational laboratory contained various options for participants to choose from, including treadmill, small trampoline, jump rope, hula hoops, free weights, video games (Nintendo Wii, Rock Band, Dance Dance Revolution), books, movies, arts and craft center, and music. At the beginning of the laboratory stay, a small saline lock intravenous catheter was placed into the participant’s forearm for subsequent blood samples, and a uniaxial accelerometer (Actigraph GT1M) was fitted on the participant’s waist for physical activity behavior assessment. For the first 5 h of each laboratory stay, the participants were interrupted every 30 min for blood samples and saliva samples, resulting in 10 blood and saliva samples for each laboratory visit. Throughout the entire 8 h laboratory stay, the participants were asked to provide ratings for their affective states. The same in-laboratory measurement procedures were implemented in the second visit.

### 2.3. Measurements

#### 2.3.1. Salivary Cortisol

Before the first meal and at every 30 min during the first 5 h of each laboratory stay, the participants were asked to put a cotton plug in their mouth until fully soaked with saliva. This data collection schedule resulted in 10 salivary cortisol samples in each visit. Each soaked cotton plug was collected in a labeled vial and stored on dry ice until the end of the laboratory visit when they were immediately transported to a freezer at –80 °C prior to the assay. Salivary cortisol levels were determined by immunometric assay on a Tosoh AIA 600II analyzer.

#### 2.3.2. Affective States

Prior to the first meal and at every 30 min during the entire 8 h of each laboratory stay, the participants were asked to rate their current affective states at the same time with serum and saliva sample collection. Five affective states were collected at each time using visual analog scales. The five-item scale included four items from the tension subscale of the Profile of Mood and States (POMS) scale [30], panic, worry, anxiousness, and nervousness, and one item for the feeling of calmness, with a possible range from 0 to 100 for each item. The average negative affective state score was calculated by averaging the scores for feelings of panic, worry, anxiousness, and nervousness at each moment. Affective state scores for each item and the average negative affective state were averaged across both visits to create personal average scores for each person. The personal average scores were then subtracted by the grand mean for the respective item to create the grand mean-centered affective state scores and herein denoted as the between-person (BP) version of the affective state scores. The BP affective state scores calculated this way represent the personal average and trait-like affective states of each participant across the study period. The within-person (WP) versions of affective states were created by subtracting the affective state scores at each measurement (i.e., every 30 min) from the respective average affective state scores for that person. The resulting scores represent the participant’s level of affective states at any given moment within the study period relative to the average level of each respective affective state of that participant. Both the BP and the WP versions will be included in the same model to disaggregate the inter- and intraindividual variabilities in outcomes of interest [31].

#### 2.3.3. Time Spent in MVPA

The amount of time spent in MVPA was assessed with a waist-worn uniaxial accelerometer (ActiGraph GT1X) across the 8 h stay for each laboratory stay. Before the beginning of the 8 h laboratory stay, an accelerometer was fitted on the participant’s right hip under the cloth using an elastic belt. The accelerometers were set to collect time-stamped data at a 60 s epoch. Accelerometer data was processed using the SAS code developed by the National Cancer Institute for use with the National Health and Nutrition Examination Survey (NHANES). The thresholds for physical activity of moderate-to-vigorous intensity were age-adjusted using the criteria by Freedson et al. [32]. The time-stamped accelerometer data were matched to the time of the affective states measurement and blood draw. Time spent in MVPA was summed for each 30 min interval. Both the BP and WP versions for time spent in MVPA were calculated using the method described in the previous section.

### 2.4. Statistical Analysis

The main outcome of interest for this study is the log-transformed cortisol value obtained at each 30 min during the laboratory stay. The rationale for using the log-transformed value is that the raw cortisol values were skewed (mean, 0.784; median, 0.680; min, 0.020; max, 41.000; skewness, 33.010; kurtosis: 1292.640) with one single extreme outlier of 41 nmol/L. After removing the extreme outlier, the distribution properties of the log-transformed cortisol values were less skewed (mean, −0.406; median, −0.386; min: −3.912; max, 1.435; skewness, −0.305; kurtosis, 1.018). This study used the log-transformed cortisol values after the removal of the one extreme outlier as the outcome variable. This study utilized multilevel modeling (MLM) to examine whether affective states are related to salivary cortisol levels in the subsequent 30 min. MLM accommodates parameter estimations that account for the non-independent nature of the outcome variables (i.e., the salivary cortisol level at each moment was nested within a laboratory visit and within a person) and allows for disaggregating the within- and between-person association between affective states and the diurnal cortisol rhythm [15,31]. Since the outcome variables were log-transformed, parameter estimates resulting from these models were interpreted after the parameter estimates were back-transformed and should be interpreted as percent changes in cortisol [14]. To investigate the moderation effects of energy balance-related behavior, interaction terms were constructed by multiplying the main predictor of the model with the moderator of interest for the model. All the final models included age, gender, and ethnicity to control for the potential confounding effects of age, gender, and ethnic differences in HPA axis activities. Analyses for this study were conducted using SAS v.9.4 (Cary, NC, USA).

## 3. Results

### 3.1. Demographic and Descriptive Statistics

Table 1 presents descriptive information on the study participants, as well as descriptive statistics of the main predictors (i.e., momentary affective state scores) and the moderator of interest (i.e., time spent in MVPA). Salivary cortisol was the highest at the beginning of the laboratory stay and declined throughout the remainder of the morning. On average, cortisol levels declined at an average rate of 16.48% per 30 min, and the rate of decline decelerated at a rate of 1.68% per 30 min throughout the morning. However, there is a significant meal type by time interaction effect (β = 0.077 (0.037), *p* < 0.05), indicating that the rate of decline differed between the two meal types when accounted for affective states. Therefore, subsequent models examined the proposed affect–HPA axis relationship separately for each meal condition.

### 3.2. Does Energy Balance-Related Behavior Moderate the Affect–HPA Axis Relationship?

When examining the affect–HPA axis relationship separately for each meal assignment, our study revealed that, at the within-person level, the average negative affective state score was associated with 1.91% (β: 0.02, *p* < 0.05) higher cortisol level 30 min after they had reported one point higher in average affective states compared to their personal average during the HS visit only (Table 2, Model 1). The average negative affective state score was not related to subsequent cortisol levels during the LS visit (Table 2, Model 2). In models with the individual item of affective state (i.e., panic, worry, nervousness, anxiousness, and calmness) as the main predictor, only levels of feeling panic were related to cortisol levels at the subsequent 30 min at the within-person level (β = 0.02, *p* < 0.01) during the HS visit (Table 3). None of the other individual affective state items were associated with levels of salivary cortisol at the subsequent 30 min and the rate of decline regardless of meal types. On the other hand, the amount of time spent in MVPA during the 30 min interval, both at the between- and the within-person level, did not moderate the relationship between affective states and subsequent cortisol levels.

## 4. Discussion

This study is one of the first to examine the relationship between fluctuations in affective states and subsequent cortisol levels and the possible moderating effects of physical activity or specific nutrient intake on this relationship in obese ethnic minority youths. The results of this study showed that negative affect was related to cortisol levels measured 30 min later, at the within-person level, and only during the high-sugar condition laboratory visit. During the HS visit, the participants were provided with breakfast that contained 41.0 g of sugar [29], a comparable level of sugar in breakfast observed in other studies conducted among urban overweight and obese ethnic minority youths [33]. The identified association in the HS condition, therefore, could represent how the obese youths’ neuroendocrine system functions after consuming breakfast on a regular day.

This study further builds on the existing literature by showing that the HPA axis is acutely reactive to the fluctuations in affective states on a momentary basis when adolescents are provided with a breakfast high in sugar content. The identified within-person process during the HS condition suggests that when adolescents experience higher-than-usual negative affective states, especially the feeling of panic, the psychological state places demands on the body, which in turn activate the HPA axis. However, while research suggests that time spent in MVPA can improve affective states in adolescents [19,20], we did not find that MVPA alleviated the effects of negative affect on the youths’ neuroendocrine system. A possible explanation for the lack of a moderating effect could be that the effect of MVPA is more pronounced in normal-weight participants, as their HPA axis reactivity differs from their obese counterparts [34]. Another potential explanation could be that participants in this study did not engage in a sufficient amount of physical activity to alleviate the effect of affective states on their neuroendocrine system. Taken together, these observed within-person associations indicate that the HPA axis is acutely reactive to both psychological demands when these demands are greater than their respective usual levels, but only after the participants consumed a breakfast with higher sugar content.

The null relationship between affective states and subsequent salivary cortisol during the LS condition is unexpected. We speculate that the possible decrease in hunger-related metabolites due to increased consumption of fiber during the LS condition may explain the absence of this acute relationship between affective states and subsequent cortisol levels. Prior research has shown that the levels of ghrelin, one of the metabolites associated with hunger, are associated with higher HPA axis reactivity [35]. Consumption of soluble fiber has also been shown to reduce the biomarkers and self-reported rating of hunger [36]. Therefore, it is possible that after consuming 16.0 g of soluble fiber during the LS condition [29], the participants’ HPA axes were less reactive to psychological and physical demands compared to the day when they were exposed to the HS condition. While this may explain the discrepancies observed within participants between an HS and LS breakfast condition, we could not examine this physiological mechanism due to the small subsample (*n* = 23) of participants with processed ghrelin assays available in the data. Other metabolites that are sensitive to the meal assignment manipulated in this study, such as leptin [37] and adiponectin [38], could also be potential sources of confounding, which could be examined in future studies. Nonetheless, the discrepant results between the HS and LS visits may suggest a possible role of sugar consumption and HPA axis reactivity.

This study had several limitations. First, while this study took place in an observation laboratory that was purposefully designed to be like a regular living room and equipped with entertainment and exercise equipment attuned to the specific participants, the youths’ emotional experiences in the naturalistic environment can still be different from those in the ambulatory and free-living environment. An important way that fluctuations in affective states captured in this naturalistic environment differ from the ambulatory environment is the absence of stressors that occur in an individual’s daily life. Therefore, future studies that examine whether the number or kind of stressors moderates the relationship between the affective states, MVPA, and subsequent cortisol levels could further elucidate the intricate relationship between the affective states, MVPA, and the HPA axis. Secondly, this study compared how the HPA axis reacts to fluctuations in affective states in two different meal type conditions, HS and LS meals. Although this study provided novel insights on the differential impact of nutrient consumption on the impact of affective states on HPA axis activation, future studies that examine the influence of insulin resistance could potentially provide more insights into how affective states acutely relate to salivary cortisol levels, as HPA axis activities are shown to be altered among those with insulin resistance [39]. Furthermore, future studies that are conducted with multiple days of specific meal conditions could yield stronger evidence for the effect of specific nutrients on the HPA axis reactivity. Third, the results of this study might not be generalizable to youths who are not overweight or not African American or Latino. Studies that include youths of other ethnic groups and of normal or low BMI would generate more insights into how racial and BMI differences are related to HPA axis reactivity and its associated mental (e.g., depression [40]) and physical (e.g., adiposity [6]) consequences.

## 5. Conclusions

In conclusion, this study showed that, among adolescents, the relationship between affective states and HPA axis activities differed when the participants were exposed to meals that differed in sugar and fiber content. While the results of this study provide novel evidence toward untangling the momentary relationship between affective states and the HPA axis, future studies with more extended observational periods and a more representative sample may yield valuable insights to whether these findings replicate in naturalistic or ambulatory settings. Furthermore, studies that examine how other meal contents attenuate the impact of negative affect on the HPA axis could potentially provide a valuable target in reducing the health risks associated with increased exposure to cortisol, including elevated risks of decreased insulin sensitivity and development of type II diabetes.

## Figures and Tables

**Table 1:** Descriptive statistics of the study participants, affective states, and time spent in MVPA.

*N* = 88	Mean	Standard Deviation
Age	16.3	1.2
***N* = 88**	**Percentage**	
Gender (%)	48.96% Male	
Ethnicity		
African American	43.18%	
Latino	56.82%	
Weight status		
Overweight (BMI percentile ≥ 85th and < 95th)	5.7%	
Obese (BMI percentile ≥ 95th)	94.3%	
**Affective States**	**Mean**	**Standard Deviation**
Feeling of panic	4.64	9.58
Feeling of anxiousness	8.79	17.48
Feeling of worry	5.48	11.48
Feeling of nervousness	6.90	14.26
Average negative affective states	6.46	11.02
Feeling of calmness	83.32	23.40
Time spent in MVPA (mins)	0.77	2.18

**Table 2:** Negative affect, time spent in MVPA, and cortisol levels by meal type.

Effect	Model 1: HS	Model 2: LS
	Estimate (SE)	Estimate (SE)
Intercept	0.713 (0.757)	−0.850 (0.796)
Negative affect (WS)	0.008 (0.003) **	−0.002 (0.003)
Negative affect (BS)	−0.005 (0.003)	−0.003 (0.004)
MVPA (WS)	0.016 (0.008) *	0.014 (0.008) ^+^
MVPA (BS)	0.073 (0.043)	0.047 (0.044)
Time	−0.148 (0.025) ***	−0.231 (0.025) ***
Time × Time	0.015 (0.002) ***	0.021 (0.002) ***

Age, gender, ethnicity, BMI percentile, and a binary variable adjusting for the order of randomization were included in all the models; + *p* < 0.10; * *p* < 0.050; ** *p* < 0.010; *** *p* < 0.001.

**Table 3:** Models of feeling panic, time spent in MVPA, and cortisol levels by meal type.

Effect	Model 1: HS	Model 2: LS
	Estimate (SE)	Estimate (SE)
Intercept	0.701 (0.762)	−0.840 (0.798)
Panic (WS)	0.007 (0.002) **	−0.003 (0.003)
Panic (BS)	−0.005 (0.005)	−0.002 (0.005)
MVPA (WS)	0.017 (0.008) *	0.014 (0.008) *
MVPA (BS)	0.071 (0.043) *	0.047 (0.044)
Time	−0.149 (0.025) ***	−0.231 (0.025) ***
Time × Time	0.015 (0.002) ***	0.021 (0.002) ***

Age, gender, ethnicity, BMI percentile, and a binary variable adjusting for order of randomization were included in all the models; * *p* < 0.050; ** *p* < 0.010; *** *p* < 0.001.

## Data Availability

Data available on request.
